# Retraction: Investigation on corrosion protection and mechanical performance of minerals substituted hydroxyapatite coating on HELCDEB-treated titanium using pulsed electrodeposition method

**DOI:** 10.1039/d3ra90027d

**Published:** 2023-03-29

**Authors:** D. Gopi, A. Karthika, D. Rajeswari, L. Kavitha, R. Pramod, Jishnu Dwivedi

**Affiliations:** a Department of Chemistry, Periyar University Salem 636011 India dhanaraj_gopi@yahoo.com +91-427-2345124 +91-427-2345766; b Centre for Nanoscience and Nanotechnology, Periyar University Salem 636011 India; c Department of Physics, School of Basic and Applied Sciences, Central University of Tamilnadu Thiruvarur 610101 India; d Industrial Accelerator Section, Raja Ramanna Centre for Advanced Technology Indore 452013 India

## Abstract

Retraction of ‘Investigation on corrosion protection and mechanical performance of minerals substituted hydroxyapatite coating on HELCDEB-treated titanium using pulsed electrodeposition method’ by D. Gopi *et al.*, RSC *Adv.*, 2014, **4**, 34751–34759. DOI: https://doi.org/10.1039/C4RA04484C.

The Royal Society of Chemistry, with the agreement of the named authors, hereby wholly retracts this *RSC Advances* article due to concerns with the reliability of the data in the published article.

Repeating fragments can be observed in the cross-sectional SEM images of Fig. 4c, f and i, indicating that the images have been manipulated.

In addition, parts of the cross-sectional SEM image in Fig. 4c have been duplicated in another publication.^1^

The authors informed the editor that the characterization of the original samples was outsourced, and they do not have the original raw data for the published results.

Given the significance of the concerns about the validity of the data, and the lack of raw data, the findings presented in this paper are not reliable.

R. Pramod and Jishnu Dwivedi were contacted but did not respond.

Signed: D. Gopi, A. Karthika, D. Rajeswari and L. Kavitha.

Date: 16th March 2023.

Retraction endorsed by Laura Fisher, Executive Editor, *RSC Advances*.

## Supplementary Material
